# Direct development in Atlantic Forest anurans: What can environmental and biotic influences explain about its evolution and occurrence?

**DOI:** 10.1371/journal.pone.0291644

**Published:** 2023-11-30

**Authors:** Rodrigo Barbosa Fontana, Camila Both, Sandra Maria Hartz

**Affiliations:** 1 Instituto de Biociências, Programa de Pós-Graduação em Ecologia, Universidade Federal do Rio Grande do Sul, Porto Alegre, Rio Grande do Sul, Brazil; 2 Departamento Interdisciplinar, Centro de Estudos Limnológicos e Marinhos, Universidade Federal do Rio Grande do Sul, Imbé, Rio Grande do Sul, Brazil; Southeastern Louisiana University, UNITED STATES

## Abstract

Different environmental and biological factors can originate and support different alternative life histories in different taxonomic groups. Likewise, these factors are important for the processes that assemble and structure communities. Amphibians, besides being highly susceptible to environmental conditions, have various reproductive strategies, such as the direct development of individuals. Several hypotheses have been raised about possible selective pressures related to the emergence of direct development in anurans, as well as the relationship between environmental characteristics and the occurrence of these species. Such investigations, however, have mainly focused on specific clades and/or regions. Here, we use structural equation modelling to investigate the relationships between different abiotic (temperature, precipitation, humidity, and terrain slope) and biotic (phylogenetic composition and functional diversity) factors and the proportion of species with direct development in 766 anuran communities of the Atlantic Forest, a biome with a vast diversity of anuran species and high environmental complexity. Anuran communities with higher proportions of direct developing species were found to be mainly influenced by low potential evapotranspiration, low temperature seasonality, and high functional diversity. Phylogenetic composition and terrain slope were also found to be important in determining the occurrence of these species in Atlantic Forest communities. These results show the importance of these factors in the structuring of these communities and provide important contributions to the knowledge of direct development in anurans.

## Introduction

As the result of a long and complex evolutionary history, the great diversity of amphibian species represents numerous morphological and behavioral varieties, an abundance of ecological relationships, and the greatest diversity of reproductive modes among tetrapods [1]. Seventy-four reproductive modes are currently described for amphibians, 56 of which are exclusive to anurans [2]. These anuran modes represent collections of reproductive traits, such as oviposition site, spawning and larval characteristics, and type of development [2,3]. Deviating from the ancestral reproductive mode of a biphasic life cycle with a larval phase in the form of a tadpole, is direct development [1,4]. Direct development in anurans is characterized by the absence of a free-living larval stage, with the hatching of miniatures of adult forms. The morphological and physiological modifications resulting from the alteration of biphasic development to a single-phase life cycle impose specific requirements and tolerances on these species, especially in terms of humidity [5,6].

Direct developing anurans are mostly associated with climatically stable environments, such as forests with higher levels of humidity and structural complexity [7–9]. In the same way that the evolutionary diversification of reproductive modes is related to distinct factors, the emergence and evolution of direct development in anurans has diverse evolutionary hypotheses, as discussed by Fontana et al. [10]. The most discussed of these hypotheses proposes direct development as an alternative trait resulting from evolutionary processes linked to the action of biotic factors, such as predation of aquatic eggs and larvae and/or competition for reproductive sites [3,11,12]. Also, the hidden amplexus of certain terrestrial breeders (some species of the families Hylidae and Leptodactylidae) may exemplify how sexual selection (competition among males for reproductive females and polyandry avoidance) molded the evolution of reproductive modes in anurans [13]. Alternatively, the abiotic environment has also been presented as a potential selective force in the evolution of direct development. Climatic conditions, such as seasonality, precipitation, and temperature, play important roles in determining the distribution of these species [4,9]. Furthermore, environmental structure, such as topography, may have also been an important selective factor, since more structurally complex locales can enhance the loss and mortality of aquatic eggs and larvae through water flow [9,14].

Continuously distributed along the Brazilian coast and penetrating inland regions, the Atlantic Forest has enormous environmental heterogeneity, with different forest formations, microclimatic conditions, and variations in relief [15,16]. Due to these characteristics, the Atlantic Forest supplies a myriad of different habitats and microhabitats that harbor an enormous diversity of plants and animals with a high rate of endemism, earning it recognition as a global biodiversity hotspot [17,18]. Among Atlantic Forest animals, the great diversity of anurans stands out, with this tropical forest sheltering almost 10% of known anuran species, placing it among the regions with the greatest diversity of this group [19,20].

The evolutionary and ecological relationships, different life histories, and great diversity of morphologies and reproductive modes of anurans are reflected not only in their high taxonomic diversity but also in their high phylogenetic and functional diversities in the Atlantic Forest [21,22]. Phylogenetic diversity (PD) is a biodiversity metric used to explore the evolutionary history of species, based on branch lengths of a phylogenetic tree [23]. Functional diversity (FD), on the other hand, not only reflects the variability of species traits in communities but can also be used as a metric to investigate complementarity in resource use, or niche differentiation, and to understand how these traits influence the functioning of communities and ecosystems [24]. Since these metrics reflect ecological and evolutionary patterns that occur in communities, they can be used to infer processes related to the response of species to biotic interactions, ecosystem functioning, and patterns related to community structure [23,25–27]. Therefore, the use of these metrics can reveal the influence of distinct factors on community assemblages, such as past competition and predation among species (in communities that present phylogenetic and/or functional overdispersion), as well as the influence of environmental filters (in communities displaying phylogenetic and/or functional clustering) [28,29]. Since biotic interactions, such as competition among species and predation, are difficult to measure in natural communities, the use of PD and FD metrics as proxies for local coexistence mechanisms and these interactions is perhaps a valid alternative.

**Fig 1:**
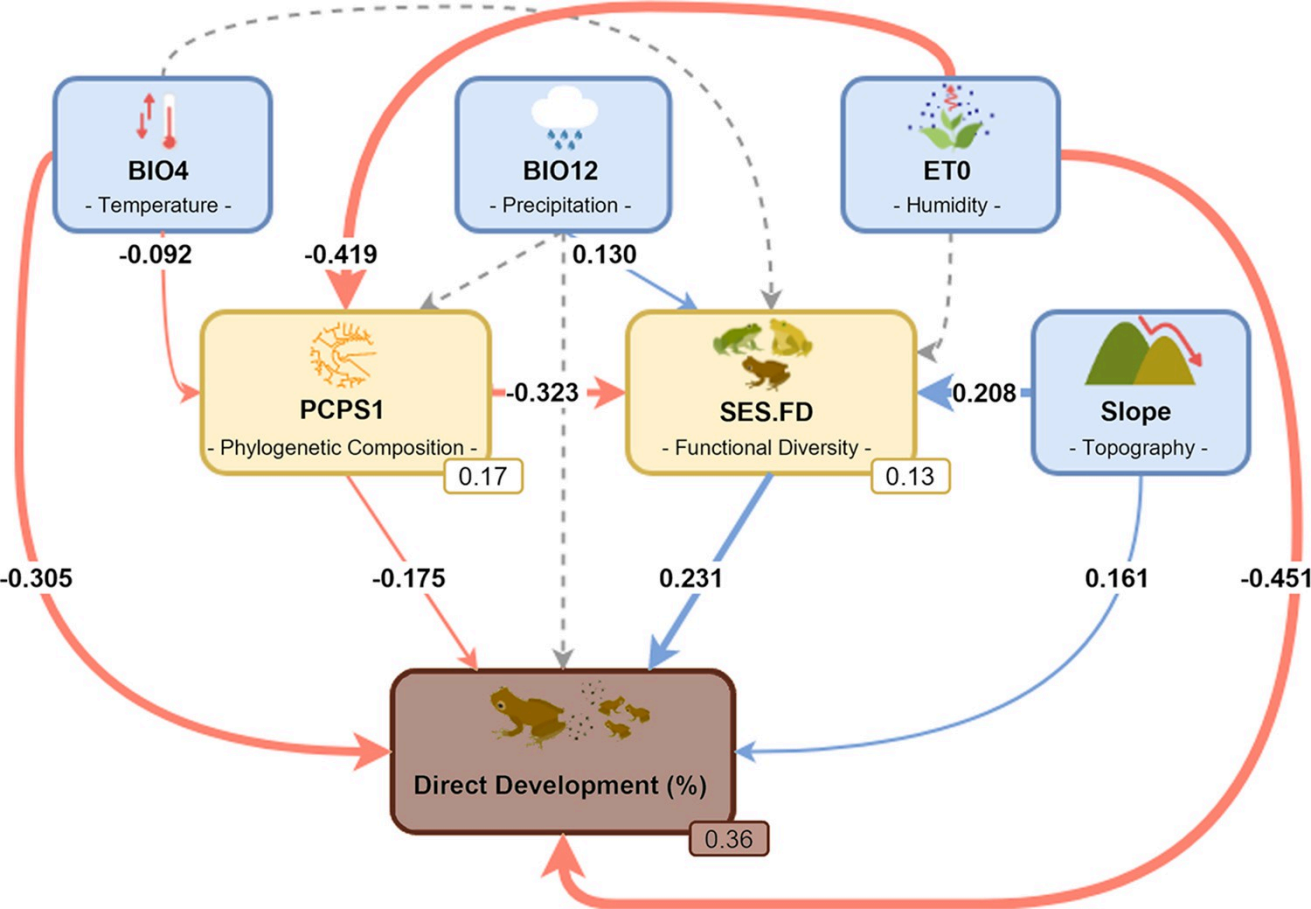
Investigated relationships of abiotic and biotic factors with direct developing anurans in the Atlantic Forest. Piecewise SEM model showing the indirect and direct effects of abiotic factors (blue boxes) and biotic factors (yellow boxes) on the proportion of direct developing species (brown box) in anuran communities of the Atlantic Forest. Significant relationships are presented in blue (positive relationship) and red (negative relationships) arrows. Values on arrows correspond to standardized coefficients. R^2^ values for endogenous variables are presented in the small boxes. The thickness of arrows is proportional to the magnitude of the standardized coefficients. BIO4 –temperature seasonality; bio12 –annual precipitation; ET0 –potential evapotranspiration; PCPS1 –first PCPS eigenvector; SES.FD–standardized effect size of functional diversity; and %DD–proportion of direct developing species in the anuran communities.

Understanding how biotic and abiotic factors are related to direct development in anurans is crucial, since these relationships can help to discover the conditions that are favorable or unfavorable for the occurrence of reproductive modes in distinct environments. Furthermore, based on these relationships, new insights can be gained into the evolution of terrestrial reproductive modes, such as direct development. Thus, here we employ structural equation modelling (SEM) to evaluate the relationships among environmental characteristics (climate and topography), biotic characteristics (phylogenetic composition and functional diversity), and the proportion of direct developing anuran species in communities of the Atlantic Forest (Fig 1).

As mentioned above, the evolution of anuran direct development can be a result of driving forces due to the actuation of abiotic factors. Thus, we expected to find higher proportions of direct developing anuran species in communities that are climatically stable and with elevated levels of humidity and rough terrain. If ecological mechanisms (e.g., competition, predation, parasitism) are also forces related to the divergence and evolution of traits, and if these mechanisms are linked to the main hypotheses regarding the evolution of direct development in anurans, we would expect that communities that experienced high levels of these interactions in the past would currently have higher levels of functional diversity and higher proportions of direct developing anuran species. In this context, communities that historically experienced high competition and predation rates must have undergone functional divergence, with species occupying new niches. As a result, these communities should currently exhibit greater functional diversity and greater proportions of direct developing anuran species.

## Material and methods

### Data collection

#### Direct developing species in Atlantic Forest anuran communities

**Fig 2:**
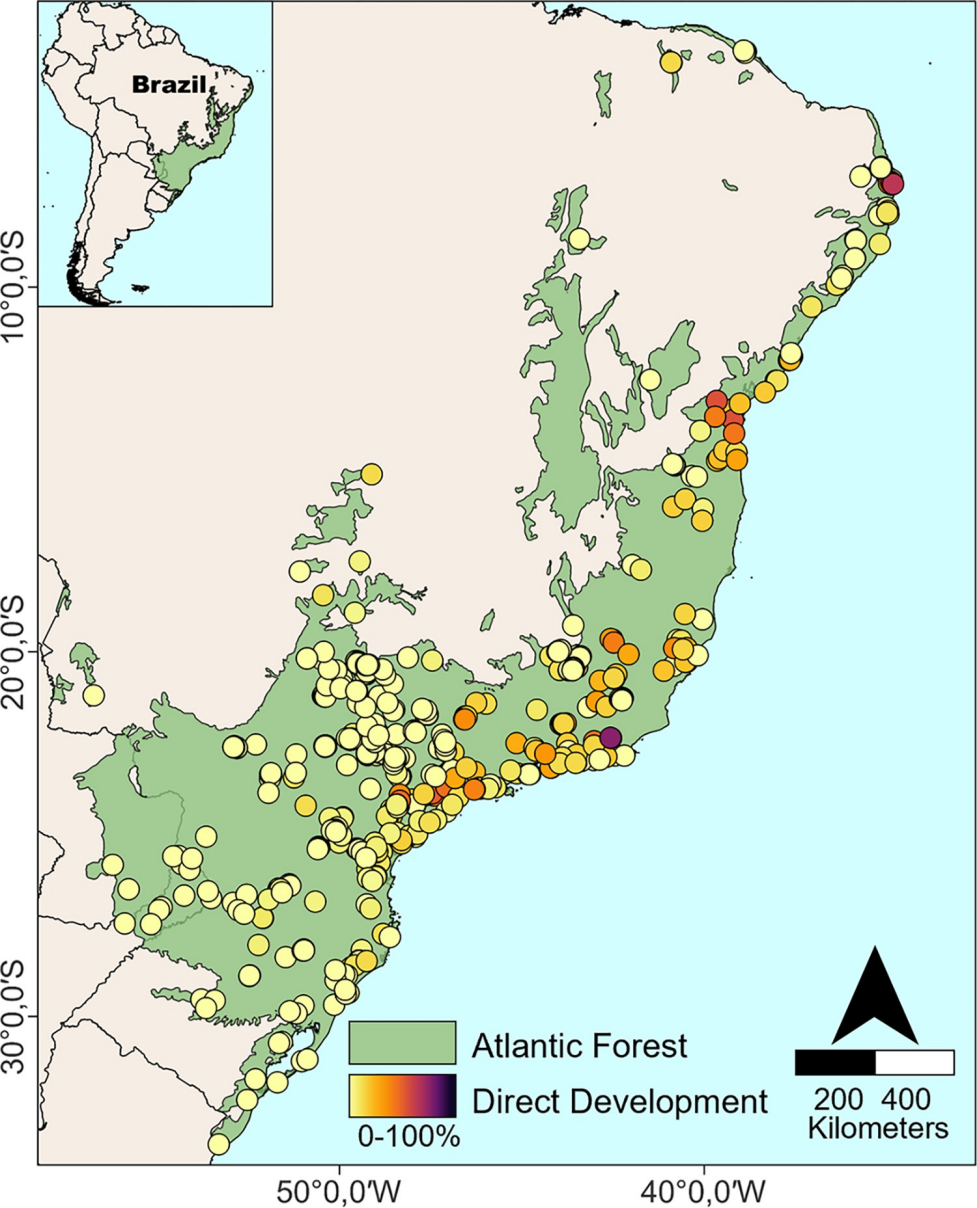
Distribution of the 766 analyzed Atlantic Forest anuran communities. The color gradient represents the observed proportion of direct developing anurans in the evaluated communities (points). Base layer sources: South America shape file retrieved from the Database of Global Administrative Areas (GADM) under an open license (CC-BY): https://gadm.org/license.html; Atlantic Forest limits reprinted from Muylaert et al. [34] under a CC BY license, with permission from Muylaert (GNU General Public License, https://github.com/LEEClab/ATLANTIC-limits/blob/master/LICENSE); communities (points) distributions were mapped based on records retrieved from Vancine et al. [30]. Modified with permission from Vancine et al. [30].

To evaluate the influences of biotic and abiotic variables on the proportion of direct developing species in Atlantic Forest anuran communities, we first created a subset of anuran communities using the database of Atlantic Forest amphibian communities of Vancine et al. [30] (S1 File). We chose to use this data set because: i) it compiles occurrence data for species that co-occur at the same place and time (study sites) distributed along the entire extension of the Atlantic Forest, thus reflecting real local communities of the region; ii) it comprises a large number of study sites (1,163), species (528) and specimen records (17,169); and iii) the data were already accurately compiled and revised. We initially prepared the data by removing all localities with missing or inaccurate geographic coordinates and those with a record of only a single anuran species. Then we calculated the observed proportion of direct developing species in each of the communities. However, as species occurrence and distribution can influence the average values of traits within communities [31], we controlled the effect of species composition on the proportion of direct developing species in the communities using null models, as proposed by Pillar and Duarte [32] and Peres-Neto et al. [33]. For this, we created a matrix containing the development type for each species present in the communities (**D matrix**). We then calculated the observed proportion of direct developing species in each community. Next, we generated a set of 999 null proportions through **D matrix** randomization and calculated the mean and standard deviation of these proportions. Finally, based on the standardized effect size metrics, we used the observed proportions and the means and standard deviations of the null proportions to calculate the proportion of direct developing species free from the composition effect for each of the communities. In total, our analyzed database comprised a comprehensive collection of 766 communities distributed along the extension of the Atlantic Forest (Fig 2).

#### Bioclimatic and topographic variables

To investigate the relationship among biotic and abiotic factors and the proportion of direct-developing anuran species in Atlantic Forest communities, we gathered bioclimatic and topographic data for each of the previously selected communities, utilizing their geographic coordinates. We downloaded the 19 bioclimatic variables from WorldClim 2.1 (https://www.worldclim.org/data/worldclim21.html) [35,36] and potential evapotranspiration (ET0) from Global Aridity Index and Potential Evapotranspiration Climate Database (https://csidotinfo.wordpress.com/2019/01/24/global-aridity-index-and-potential-evapotranspiration-climate-database-v3/) [37]. We considered terrain slope as the topographic variable, which represents the gradient of change in terrain elevation and is related to water accumulation and the direction and velocity of water flow [38]. Thus, slope reflects important aspects of terrain topography that can favor or hinder oviposition and survival of anuran offspring in the environment, such as waterbody availability and water flow velocity. This measure was downloaded from the EarthEnv database (www.earthenv.org) [38]. After downloading the data, we cropped all the rasters to match the extent of the Atlantic Forest boundaries [34], to obtain the bioclimatic and topographic values for each selected community. All rasters were downloaded at a resolution of 30 arc-seg (approximately 1km at the equator).

To reduce the number of abiotic variables in our final model, we initially fitted two linear models using the proportion of direct development species in each community as the response variable and abiotic variables (temperature and precipitation) as predictors. We then selected models based on the Akaike information criterion (AIC) [39]. The AIC ranks a given set of distinct candidate models based on their respective goodness-of-fit to data and the associated model complexity, reflecting the amount of information loss. Consequently, models with lower AIC values are deemed the most appropriate, representing an optimized balance between accurate data fitting and model simplicity [39]. We used stats and MuMIn packages of the R environment [40,41] to perform the fitting and selection of models. After model selection, and due to the importance of these variables to the occurrence and distribution of anurans [42–45] (S2 File), we opted to use the following variables in our model: temperature seasonality (BIO4)–representing temperature; annual precipitation (BIO12)–representing precipitation; potential evapotranspiration–representing humidity; and terrain slope–representing topography.

#### Phylogenetic composition

To consider the evolutionary history of the assemblages and to determine its role in the occurrence of direct developing species in Atlantic Forest anuran communities, we opted to incorporate phylogenetic composition in our final model. To do this, we created a phylogenetic tree of our species pool (subtree) using as a base the consensus phylogenetic hypothesis proposed by Jetz and Pyron [46]. We used the geiger package [47] to prune and check the correspondence between the species pool of our data set and the consensus phylogenetic hypothesis and identified the absence of eight species, also referred to as phylogenetically uncertain taxa (PUTs), namely: *Chiasmocleis lacrimae; Eleutherodactylus bilineatus; Boana cambui*, *Phrynomedusa dryade; Phyllomedusa rustica; Proceratophrys mantiqueira; Scinax melanodactylus; and Trachycephalus typhonius*. We included these PUTs in the subtree by using information from the literature to define the most derived consensus clade (MDCC), which represents the most basal node containing the closest living relative [for more details see 48]. We added the PUTs to our subtree (S1 Appendix) using PAM v0.9 software [48]. We then used a principal coordinates of phylogenetic structure (PCPS) analysis, a method based on a principal coordinates analysis (PCoA) of a matrix containing phylogeny-weighted species composition (**P** matrix), which produces a series of eigenvectors that describe orthogonal gradients of phylogenetic structure [49,50]. Whereas the first PCPS eigenvector (with higher eigenvalues) captures deeper relationships in the phylogeny (basal nodes of the phylogenetic tree), subsequent PCPS eigenvectors describe the relationships between more recent lineages (terminal nodes) [51]. We used the PCPS package [52] in the R environment to obtain phylogenetic composition [47].

#### Functional diversity

To calculate FD, we initially compiled information about reproductive and morphological traits using data sets, articles and books to create a database containing the following seven functional traits: i) oviposition type (two categories: aquatic or terrestrial); ii) development type (two categories: larval or direct); iii) juvenile habitat (three categories: terrestrial, aquatic, or semiterrestrial–the last for those that inhabit and occupy both environments); iv) oviposition site (seven categories: eggs directly into water or submersed substrate; eggs in bubble nests on still water; eggs in foam nests; eggs embedded in back of aquatic female; eggs on ground, rocks or in burrows; arboreal eggs attached on plants; or eggs carried by terrestrial adults); v) body length; vi) head width; and vii) tibia length (S3 File). We used mean values for morphological traits whenever they were accessible in the literature, but in cases where only maximum values were available, those were considered. We chose these traits because they can be related to resource use complementarity, reproductive niche differentiation, and habitat use [53,54], and thus are able to indicate possible effects of species co-occurrence and interspecific competition.

Despite the effort, we were not able to collect information about head width for 139 species and tibia length for 146 species. Therefore, for species with missing data, we inferred these traits through a maximum likelihood approach using the phylogenetic tree and the Rphylopars package [55]. This approach of trait inference assumes that the traits are correlated with each other, and with phylogenetic relationships among species [55,56]. We also evaluated possible differences in the analyses that excluded traits associated with direct development, since the use of these traits could indicate a circular relationship with the proportion of direct developing species in communities. After finding no differences, we decided to retain these traits in our analysis. After creating the trait matrix, we performed a phylogenetic eigenvector regression (PVR) using the daee package [57] to control the effects of phylogenetic autocorrelation on functional traits. PVR is based on calculating a phylogenetic distance matrix followed by performing a PCoA, which produces a series of orthogonal eigenvectors that are independent of phylogenetic structure [58].

Finally, to reduce the effect of species number on functional diversity in the Atlantic Forest amphibian communities and to allow comparisons of communities with different richness values, we calculated the standardized effect size of functional diversity (SES.FD) [59]. These metrics are based on null model analyses and allow the removal of possible biases related to species richness when considering both observed values and values generated by null models and can also reveal phylogenetic or functional patterns of community structure [59]. In this case, while communities structured by biotic factors, such as competition and predation, will present functional overdispersion (identified by positive values of SES.FD), communities strongly influenced by abiotic factors, such as environmental filters, will present functional clustering (identified by negative values of SES.FD) [60–62].

To calculate SES.FD we generated a distance matrix based on the first 283 phylogenetic eigenvectors (which contained 95% of data variation) previously obtained from the PVR analysis, which were later transformed into a dendrogram using the stats package [41]. Then, based on the dendrogram, we calculated the observed functional diversity of Petchey and Gaston 2002 [25,63], a metric that is derived from the phylogenetic diversity of Faith [23] and is based on the sum of branch lengths of a functional dendrogram. To calculate SES.FD we used as the null model the permutation of the species on the terminal nodes of the dendrogram (taxa.labels), 999 randomizations and the picante package [64].

### Data analyses

To evaluate the relationships among biotic and abiotic variables and the proportion of direct developing species in Atlantic Forest anuran communities, as proposed in Fig 1, we used SEM by piecewise [65]. The use of SEM models enables us to investigate and confirm direct and indirect causal relationships, proposed from a conceptual model, which is generated a priori based on specific knowledge of the studied system, while piecewise allows us to incorporate random effects and autocorrelation structures in the models [65,66]. We used the first PCPS eigenvector (PCPS 1) and the standardized effect size of functional diversity (SES.FD) as indicative of biotic factors. We chose these metrics because they allow us to investigate the effect of evolutionary history and aspects related to species co-occurrence. For the abiotic factors, we used temperature seasonality (BIO4) (as an indicator of temperature), annual precipitation (BIO12) (as an indicator of precipitation), potential evapotranspiration (ET0) (as an indicator of air humidity), and slope (as an indicator of topography).

Prior to fitting the SEM model, we tested and verified the absence of spatial autocorrelation in the data. To do this, we constructed generalized least squares models (GLS) containing the abiotic and biotic variables, and the proportion of direct developing species (S4 File) with exponential autocorrelation structure using the geographic coordinates of each community (latitude and longitude) using the nlme package [67]. Finally, we used these GLS models to generate our final SEM model through the piecewiseSEM package [65]. We considered SEM model validity through statistical significance (p > 0.05). All variables were previously standardized, and the analyses were performed in R [41].

## Results

**Fig 3:**
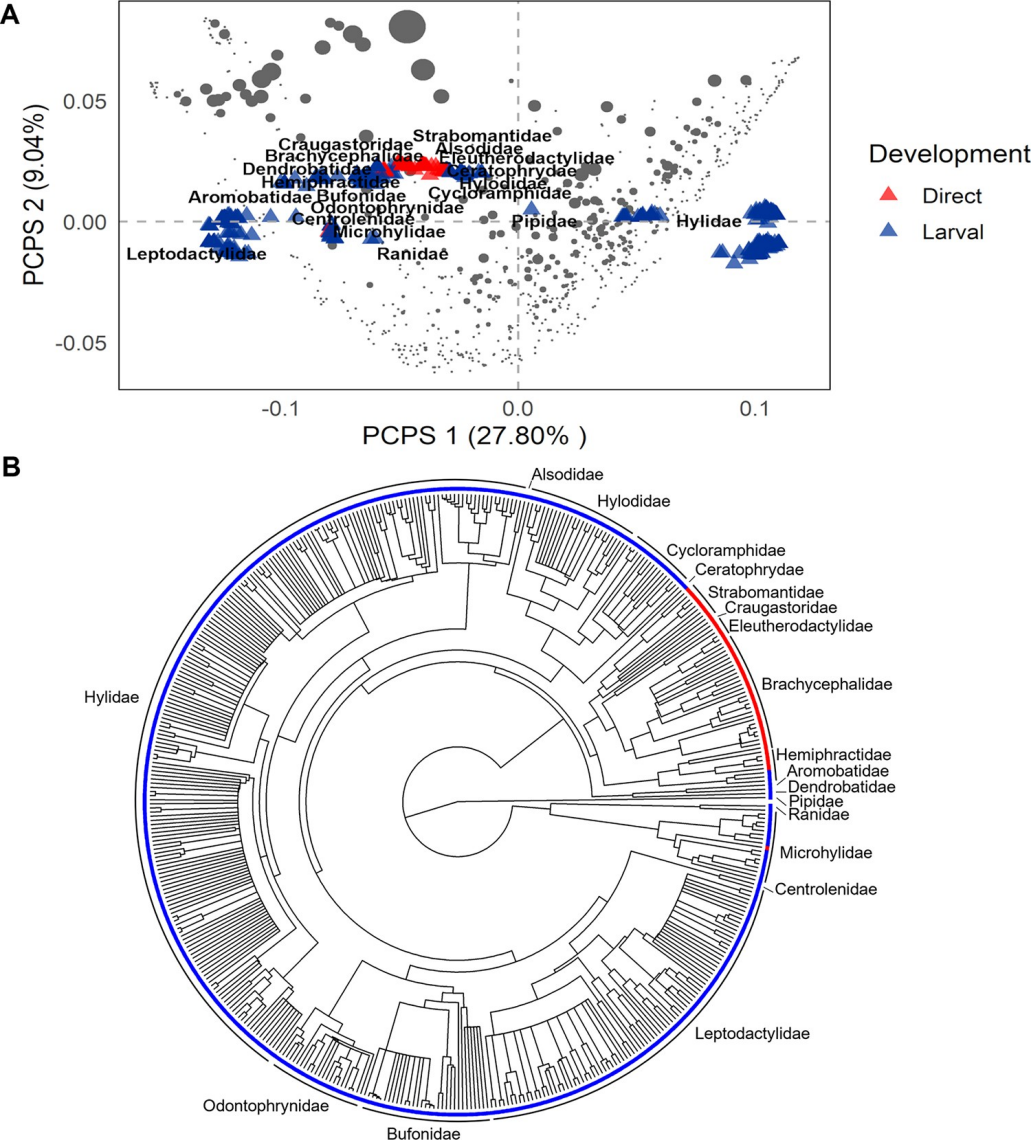
Direct development and Atlantic Forest anurans. (A) Phylogenetic distribution of direct development in 464 Atlantic Forest anurans. Families outlined in blue comprise species with biphasic development, while families highlighted in red encompass species with direct development. Species relationships were based on the consensus phylogenetic tree of Jetz and Pyron [46]. For a more detailed version with species names, see S2 Appendix. (B) Scatter plot of PCPS eigenvectors 1 and 2 generated for anurans occurring in Atlantic Forest communities. Gray circle size indicates the proportion of direct developing species in the communities, while red and blue triangles represent direct development larval development, respectively.

Our full dataset comprised 464 species of 19 families distributed among 766 anuran communities along the full extension of the Atlantic Forest, with species richness ranging from 2 to 49 species per community (mean = 13.48, SD = 9.17). Just over 10% (49) of the recorded species had a reproductive mode with direct development, and they were concentrated in a few lineages (Fig 3A, see more detailed in S2 Appendix). About 31% (237) of the evaluated communities contained species with direct development, with the others being exclusively composed of species with larval development. The proportion of direct-developing anurans in the communities ranged from 0 to 100% (mean = 4.32%, SD = 9.23), with a maximum richness of 10 species with direct development within a community. Communities with higher proportions of direct developing species were found spatially clustered in the southern Atlantic Forest and penetrating some areas in the northern Atlantic Forest (Fig 2).

Together the first two PCPS eigenvectors captured 36.84% of the total variation in the phylogenetic composition data. Eigenvector 1 split communities that were formed by hylids with lower proportions of direct developing anurans from those formed by aquatic and terrestrial species (other lineages) with higher proportions of direct developing species. In turn, PCPS 2 was positively related to communities formed by terrestrial species with higher proportions of direct developing species and negatively related to communities formed by species with aquatic reproductive modes (Leptodactylidae, Microhylidae and Ranidae, Fig 3B).

Our model, which correlates the selected climatic and topographic variables, phylogenetic composition, and functional diversity with the proportion of direct-developing species in Atlantic Forest anuran communities, explained 36% of the variation in the data (Fischer’s C_2_ = 2.635, p = 0.268, Fig 1, and S5 File). Potential evapotranspiration was the principal factor explaining the proportion of direct developing species, being negatively related (r = -0.451), followed by temperature seasonality with a negative effect (r = -0.305) and functional diversity with a positive effect (r = 0.231). Only annual precipitation had no direct effect on the proportion of direct developing anurans; however, it had a small effect on functional diversity. Also, climate and topography together explained 17% of the variation in phylogenetic composition and 13% of the variation in functional diversity (SES.FD) (Fig 1, and S5 File).

The communities with the highest proportions of direct developing anurans were distributed in regions with low to moderate temperature seasonality, low potential evapotranspiration and steeply sloping topography (see S1 Fig in S3 Appendix). Although we could not find any significant pattern of functional structural in the vast majority of the studied communities, southern Atlantic Forest communities showed negative values of SES.FD, indicating functionally similar species (functional clustering; see S2 Fig in S3 Appendix). Conversely, a few communities in the northern Atlantic Forest presented positive values of SES.FD, which suggests that these communities are composed of functionally distinct species (functional overdispersion) (S2 Fig in S3 Appendix).

## Discussion

### Direct development in Atlantic Forest anuran communities

Atlantic Forest anuran communities are composed of a variety of species with distinct evolutionary histories, physiological requirements, and reproductive strategies. The analyzed species pool included about 65% of the 719 anuran species currently known for the Atlantic Forest [20]. Our results showed that most of the communities are composed of species with larval development, which is considered ancestral and phylogenetically predominant among amphibians [1,4,68]. Ten-percent of the anuran species considered in the present study have direct development as a reproductive strategy, whereas estimates at the global level indicate about 25% [69]. At least a third of the described species in the Neotropical region have direct development, with Central American forests being the main refuge of these species, followed by the Atlantic Forest [4,70,71]. Although direct development is widespread throughout the phylogeny of amphibians and has evolved multiple times in different lineages [4], in the Atlantic Forest this reproductive strategy is concentrated in certain families and genera, such as the clade Terrarana and some species of the family Hemiphractidae [19,72].

The clade Terrarana is a species complex, formed by four families of direct developing anurans (Brachycephalidae, Craugastoridae, Eleutherodactylidae, and Strabomantidae), distributed throughout the New World (North, Central, and South America and West Indies) [72]. It emerged in South America during the Cenozoic and has since dispersed and radiated to the other regions [72,73]. In addition to terrarans and some hemiphractids, the phylogenetic tree of anurans of the Atlantic Forest reveals the presence of another clade with a single species representing direct development (*Myersiella microps*). The finding that almost all direct developers of the Atlantic Forest belong to only one clade supports the idea that South America served as a center of origin for this group of species. It also supports the idea that past environmental conditions of the coastal portion of the Atlantic Forest restricted the dispersal and occurrence of certain lineages to a suitable ecological niche [7,73,74], such as the case for Brachycephalidae. The family Brachycephalidae, represented by the genera *Brachycephalus* and *Ischnocnema*, is the most represented lineage of direct developers in the Atlantic Forest. It is associated with montane forests, with a distribution restricted mainly to southern and southeastern portions of the Atlantic Forest [72,75,76]. However, the present results indicated that the proportion of direct developers in the communities is not just a mere reflection of the center of origin of these few clades in the Atlantic Forest, but is also a result of selective forces, such those due to biotic and abiotic factors.

### Biotic factors and direct development in Atlantic Forest anuran communities

The effect of biotic factors on the evolution of direct development has never been effectively evaluated at the community scale, only by species-specific experiments. In this way, when functional diversity was used as a proxy of co-occurrence mechanisms, the model showed a significant positive relationship between this metric and the proportion of direct developing species in Atlantic Forest anuran communities. Thus, our results indicated that communities with higher functional diversity (and, therefore, increased likelihood of all kinds of biotic interactions such as competition and predation) were formed by a higher proportion of direct developing species, suggesting that direct development evolved as an alternative to minimize and/or avoid biotic pressures, as the main evolutionary hypotheses proposed [10,11,77]. Furthermore, a scenario can be envisioned in which even without these interactions, the evolution of alternative reproductive modes by pure neutral mechanisms can be favored by enhanced functional diversity of other functional traits. In this context, when species arrive in new environments, those with more specialized reproductive modes that deviate from the classical aquatic reproductive modes, such as direct developers, will be able to occupy new niches [1,68,78], avoiding the inhibitions imposed by priority effects.

Patterns of phylogenetic and/or functional structure of communities can be identified at different scales, reflecting the actuation of distinct processes in their formation [79]. Paz et al. [62] found some communities in extreme environments of the Atlantic Forest to be functionally clustered, suggesting that environmental filters played a significant role in assembling them [28,60,62]. Communities that are strongly influenced by environmental conditions can be molded and structured by the selection of traits related to high competitive capacity of species [80]. The structural model developed here aimed to provide the best alternative to assessing how distinct factors influenced community structure. However, functional clustering or overdispersion patterns were not detected in the majority of the studied anuran communities. Consequently, our results, at the community scale, indicated that stochastic processes were probably more critical for community assembly [28,81]. Nevertheless, it is important to point out that only a few traits that could be assessed were explored. Therefore, the perceived absence of a significant pattern of functional structure in most communities may in fact be a consequence of stochastic processes, as mentioned above, but also a result of other aspects not explored here since results can vary according to the selected functional traits [82]. Some examples of other traits are those related to movement, reproduction, and habitat use/selection, such as migration and dispersion movements, sexual dimorphism and number of eggs or offspring, and habitat preference, respectively [53].

### Abiotic factors and direct development in Atlantic Forest anuran communities

As expected, we verified that the proportion of direct developing species in Atlantic Forest anuran communities is related to both biotic factors, namely phylogenetic composition and functional diversity, and abiotic factors, namely climate and topography. The different responses of amphibians to climatic conditions are known to be mediated by their reproductive modes [83]. Reproductive specialization can be considered not only a result of the phylogenetic relationships among families but also as a response to the environmental conditions of the region in which the species evolved [1,3,8]. In this sense, Lourenço-de-Moraes et al. [84], showed that the high richness and endemism rates of amphibians in the Atlantic Forest can be explained by climatic conditions, such as annual mean temperature and annual precipitation. Similarly, environmental factors, such as climate and environmental structure, can also define and delimit the occurrence and proportion of amphibians with terrestrial reproduction in communities [4,8,9].

Historically, the climatic seasonality of the coastal region of the Atlantic Forest delimited and restricted the occurrence of species with specialized reproductive modes to this portion that is climatically more stable [7]. Amphibians from the tropics tend possess a narrower amplitude in the temperature dimension of their niche due to low-temperature seasonality in the region [43]. In this context, the negative relationship found between the proportion of direct developing species in Atlantic Forest anuran communities and temperature seasonality reinforces the idea that climatic conditions must have influenced the evolution of direct development [4]. It is also important to highlight that montane environments in the coastal region have not only provided suitable conditions for the occurrence of species with distinct life histories, but also currently condense the largest forest remnants of the Atlantic Forest [7,85]. Therefore, the montane forests function as important refuges for the conservation of amphibian functional diversity, since species with specialized reproductive modes, such as direct development, are highly susceptible to environmental changes [21,86–88].

Although terrestrial direct developers show high independence from water bodies, they remain dependent on humidity in the environment [77]. In the same way that climatic oscillations restricted the expansion of the distributions of species with specialized reproductive modes, the high humidity level of the Atlantic Forest favored the high phylogenetic and reproductive diversity found in the region [7,42]. The importance of annual precipitation to the richness, occurrence, and distribution of anurans has already been demonstrated by several studies undertaken in different regions [89–92]. Ochoa-Ochoa et al. [92] found that amphibian functional diversity is influenced by climate, with humid mountain regions being the most diverse due to high annual precipitation and low precipitation seasonality. Here, we did not find a direct effect of annual precipitation on the proportion of direct developing species in the studied Atlantic Forest anuran communities, although we observed a negative relationship between this proportion and potential evapotranspiration. Nonetheless, these results do not necessarily reject the importance of these variables to these anurans, since we verified an indirect effect of annual precipitation on the occurrence of direct developing species mediated by functional diversity. Similarly, when considering spatial autocorrelation, Gimenez and Vasconcelos [93] also did not find a correlation between the diversity of terrestrial reproductive modes (non-aquatic eggs) and annual precipitation and evapotranspiration in the Atlantic Forest but verified that these reproductive modes are related to the presence of ombrophilous forests (evergreen). Thus, the water and humidity requirements of anurans, especially terrestrial breeders (including direct developers), might be supplied by specific conditions of humid microhabitats that are only perceived at finer, and not larger, spatial scales.

Another factor that is also considered to promote reproductive diversification and specialization is the complex topography of the Atlantic Forest [3]. High altitude regions are known to possess higher topographic heterogeneity, which directly influences biodiversity [94,95]. Among the different topographic measures, slope is related to the direction and velocity of water flow, as well as water accumulation, which exercises distinct pressures on organisms [38]. In environments with high slopes, the velocity of water flow tends to be more accentuated, which may affect the different organisms that occupy these environments and increase the mortality of non-adapted species [96]. In this sense, some studies have mentioned terrain topography as a potential driving force in the evolution of terrestrial reproduction and direct development. Liedtke et al. [9] verified a strong relationship between high slope environments and the occurrence of terrestrial breeders (including those with direct development), whereas Portik et al. [97] did not detect co-evolutionary relationships between terrestrial oviposition sites and lotic environments. Whereas our results do not effectively confirm this hypothesis, they do suggest it, since we observed a higher proportion of direct developing anurans in localities with greater slopes.

### Contributions, perspectives and future directions of knowledge of anuran direct development

Despite the important relationships observed among the climatic conditions, topography, biotic factors, and the proportion of direct development species in the studied Atlantic Forest anuran communities, a significant part of our data remained unexplained. In this context, other factors that we did not evaluate here could also be related to the structuring of these communities, such as micro-environmental and biogeographical factors. As noted by Lion et al. [45], environmental variables can act in diverse ways on the occurrence and proportion of terrestrial-breeding amphibians in communities depending on the biogeographic region that is considered. Since Lutz [11] spotlighted the probable factors related to the evolution of direct development in amphibians, several other authors have been investigating and testing these hypotheses but focusing on species-specific experiments, and/or factors independently. Here, we are proposing an integrated way to explore the relationships among distinct factors (biotic and abiotic) and direct development in anurans. We recognize that our model does not encompass other different factors and sources of variation. Nevertheless, it does indeed provide a meaningful framework for understanding the important mechanisms influencing real biological communities. Thus, future analyses comparing the relationships among abiotic and biotic factors and direct development in anurans performed at different scales and in other regions, and thus encompassing distinct species pools, more clades and greater climatic and environmental variability, will be able to show distinct patterns among these relationships and help to amplify knowledge about this specialized reproductive mode.

Direct developers have particular life history and ecological traits, such as an association with high-altitude environments, restricted distributions, and smaller clutches with larger eggs, traits that can be associated with extinction risk [5,71,98–101]. As verified here, the occurrence of direct developing species of Atlantic Forest anuran communities was mostly associated with temperature seasonality and potential evapotranspiration, hence, future climatic changes can impoverish these communities, especially by the loss of this group of species. Additionally, the use of information about life-history traits, such as the developmental mode of anurans, can be useful to indicate priority areas for conservation efforts [102]. In this sense, we also highlight the importance of conservation plans and actions specific to these species, as well as to high-altitude forests, which are true refuges for direct developing anurans, to ensure the maintenance and conservation of different anuran evolutionary and life histories. Moreover, the observed relationships among abiotic and biotic variables and the proportion of direct developing anurans can also help to reinforce the hypothesis that multiple selective forces guided the evolution of this reproductive mode. Finally, we emphasize the importance of our results as one of the few studies that effectively aimed to relate anuran direct development with distinct factors on a wide spatial scale, independently of specific lineages and families, and involving many evaluated communities.

## Supporting information

S1 File Atlantic Forest anuran communities. Subset of 766 Atlantic Forest anuran communities based on Vancine et al. 2018 [30] data.
(XLSX)

S2 File Model selection used for temperature and precipitation variable selection. (DOCX)

S3 File Dataset of life-history traits of Atlantic Forest anurans. (XLSX)

S4 File Data of Atlantic Forest anuran communities. Biotic and abiotic factors, and the proportion of direct development species of 766 Atlantic Forest anuran communities. *Standardized data.
(XLSX)

S5 File Result of piecewiseSEM model. Direct and indirect effects of predictor variables on proportion of direct developing species of Atlantic Forest anuran communities.
(DOCX)

S1 Appendix Phylogenetic tree of the Atlantic Forest anurans. (TXT)

S2 Appendix Phylogenetic distribution of the development mode of 464 Atlantic Forest anurans. (PDF)

S3 Appendix Distribution of Atlantic Forest anuran communities and environmental variables. (DOCX)
