# Supplementary material for: Direct development in Atlantic Forest anurans: What can environmental and biotic influences explain about its evolution and occurrence?
Source: PLoS One. 2023 Nov 30;18(11):e0291644. doi: 10.1371/journal.pone.0291644 (PMC10688756; doi:10.1371/journal.pone.0291644)
Supplement: S2 File — (DOCX) [file pone.0291644.s002.docx]

**S1 File – Model selection used for temperature and precipitation variable selection.**

| **Model selection: Temperature variables** | | | | | | | | | | | | | | | | | | | | | | | | | | | | |  | |  |  |
| --- | --- | --- | --- | --- | --- | --- | --- | --- | --- | --- | --- | --- | --- | --- | --- | --- | --- | --- | --- | --- | --- | --- | --- | --- | --- | --- | --- | --- | --- | --- | --- | --- |
| Components | | | GL | | | | logLik | | | | | | AICc | | | | delta | | | weight | | | |  | |  | | |  | |  |  |
| 1,2,4,6,7 | | | 7 | | | | -1006.10 | | | | | | 2026.35 | | | | 0 | | | 0.15 | | | |  | |  | | |  | |  |  |
| 1,2,4,6,7,9 | | | 8 | | | | -1005.42 | | | | | | 2027.04 | | | | 0.69 | | | 0.11 | | | |  | |  | | |  | |  |  |
| 2,3,4,5,6,7 | | | 8 | | | | -1005.46 | | | | | | 2027.11 | | | | 0.77 | | | 0.10 | | | |  | |  | | |  | |  |  |
| 1,2,4,6,7,8 | | | 8 | | | | -1005.50 | | | | | | 2027.20 | | | | 0.85 | | | 0.10 | | | |  | |  | | |  | |  |  |
| 1,2,3,4,6,7 | | | 8 | | | | -1005.61 | | | | | | 2027.40 | | | | 1.06 | | | 0.09 | | | |  | |  | | |  | |  |  |
| 1,2,4,5,6,7 | | | 8 | | | | -1005.62 | | | | | | 2027.43 | | | | 1.08 | | | 0.09 | | | |  | |  | | |  | |  |  |
| 2,3,4,6,7 | | | 7 | | | | -1006.64 | | | | | | 2027.43 | | | | 1.08 | | | 0.09 | | | |  | |  | | |  | |  |  |
| 2,3,4,6,7,8 | | | 8 | | | | -1005.78 | | | | | | 2027.76 | | | | 1.41 | | | 0.08 | | | |  | |  | | |  | |  |  |
| 1,2,3,4,6,7,8 | | | 9 | | | | -1004.93 | | | | | | 2028.10 | | | | 1.75 | | | 0.06 | | | |  | |  | | |  | |  |  |
| 1,2,3,4,5,6,7 | | | 9 | | | | -1004.93 | | | | | | 2028.11 | | | | 1.76 | | | 0.06 | | | |  | |  | | |  | |  |  |
| 1,2,3,4,6,7,9 | | | 9 | | | | -1004.94 | | | | | | 2028.13 | | | | 1.78 | | | 0.06 | | | |  | |  | | |  | |  |  |
| Term codes | | |  | | | |  | | | | | |  | | | |  | | | | | | |  | | | | |  | |  |  |
| BIO1 | BIO10 | | | BIO11 | BIO2 | | | BIO3 | | | BIO4 | | | BIO7 | | BIO8 | | BIO9 | | |  | | | |  | | |  |  |  |  |  |
| 1 | 2 | | | 3 | 4 | | | 5 | | | 6 | | | 7 | | 8 | | 9 | | |  | | | |  | | |  |  |  |  |  |
| Model-averaged coefficients | | | | | | | | | | | | |  | | | |  | | |  | | | |  | |  | | |  | |  |  |
| (full average) | | | | | | |  | | | | | |  | | | |  | | |  | | | |  | |  | | |  | |  |  |
|  | | | Estimate | | | | S.E | | | | | | Adj. Error | | | | z value | | | Pr(>\|z\|) | | | |  | |  | | |  | |  |  |
| Intercept | | | 1.76E-15 | | | | 3.26E-02 | | | | | | 3.26E-02 | | | | 0.000 | | | 1.000 | | | |  | |  | | | | |  |  |
| **BIO1** | | | **-1.05E+00** | | | | **8.87E-01** | | | | | | **8.87E-01** | | | | **1.181** | | | **0.237** | | | |  | |  | | | | |  |  |
| **BIO10** | | | **1.76E+00** | | | | **8.95E-01** | | | | | | **8.96E-01** | | | | **1.968** | | | **0.049** | | | |  | |  | | | | |  |  |
| **BIO2** | | | **-1.07E+00** | | | | **3.38E-01** | | | | | | **3.38E-01** | | | | **3.167** | | | **0.001** | | | |  | |  | | | | |  |  |
| **BIO4** | | | **-2.04E+00** | | | | **7.17E-01** | | | | | | **7.17E-01** | | | | **2.841** | | | **0.004** | | | |  | |  | | | | |  |  |
| **BIO7** | | | **1.30E+00** | | | | **3.97E-01** | | | | | | **3.97E-01** | | | | **3.282** | | | **0.001** | | | |  | |  | | | | |  |  |
| BIO9 | | | 2.20E-02 | | | | 6.69E-02 | | | | | | 6.69E-02 | | | | 0.329 | | | 0.742 | | | |  | |  | | | | |  |  |
| BIO11 | | | -1.47E+00 | | | | 1.86E+00 | | | | | | 1.86E+00 | | | | 0.791 | | | 0.429 | | | |  | |  | | |  | |  |  |
| BIO3 | | | 6.79E-02 | | | | 1.60E-01 | | | | | | 1.60E-01 | | | | 0.424 | | | 0.671 | | | |  | |  | | |  | |  |  |
| BIO8 | | | -1.72E-02 | | | | 4.31E-02 | | | | | | 4.31E-02 | | | | 0.400 | | | 0.689 | | | |  | |  | | |  | |  |  |
| (conditional average) | | | | | | |  | | | | | |  | | | | | | |  | | | | | |  | | | | |  |  |
|  | | | Estimate | | | | S.E | | | | | | Adj. Error | | | | z value | | | Pr(>\|z\|) | | | |  | |  | | |  | |  |  |
| Intercept | | | 1.77E-15 | | | | 3.26E-02 | | | | | | 3.26E-02 | | | | 0 | | | 1 | | | |  | |  | | |  | |  |  |
| **BIO1** | | | **-1.43E+00** | | | | **7.24E-01** | | | | | | **7.25E-01** | | | | **1.978** | | | **0.047** | | | |  | |  | | |  | |  |  |
| **BIO10** | | | **1.76E+00** | | | | **8.95E-01** | | | | | | **8.96E-01** | | | | **1.968** | | | **0.049** | | | |  | |  | | |  | |  |  |
| **BIO2** | | | **-1.07E+00** | | | | **3.38E-01** | | | | | | **3.38E-01** | | | | **3.167** | | | **0.001** | | | |  | |  | | |  | |  |  |
| **BIO4** | | | **-2.04E+00** | | | | **7.17E-01** | | | | | | **7.17E-01** | | | | **2.841** | | | **0.004** | | | |  | |  | | |  | |  |  |
| **BIO7** | | | **1.30E+00** | | | | **3.97E-01** | | | | | | **3.97E-01** | | | | **3.282** | | | **0.001** | | | |  | |  | | |  | |  |  |
| BIO9 | | | 1.28E-01 | | | | 1.11E-01 | | | | | | 1.12E-01 | | | | 1.150 | | | 0.250 | | | |  | |  | | |  | |  |  |
| BIO11 | | | -2.68E+00 | | | | 1.75E+00 | | | | | | 1.75E+00 | | | | 1.529 | | | 0.126 | | | |  | |  | | |  | |  |  |
| BIO3 | | | 2.64E-01 | | | | 2.18E-01 | | | | | | 2.18E-01 | | | | 1.209 | | | 0.226 | | | |  | |  | | |  | |  |  |
| BIO8 | | | -7.21E-02 | | | | 6.17E-02 | | | | | | 6.18E-02 | | | | 1.167 | | | 0.243 | | | |  | |  | | |  | |  |  |
| **Model selection: Precipitation variables** | | | | | | | | | | | | | | | | | | | | | | | | | | | | | |  |  |  |
| Components | | GL | | | | logLik | | | AICc | | | | | | delta | | | | weight | | |  | | | | |  | | |  |  |  |
| 1,2,3,4,6,7,8 | | 9 | | | | -1028.07 | | | 2074.37 | | | | | | 0 | | | | 0.30 | | | |  | | | |  | | |  |  |  |
| 1,2,3,4,6,7 | | 8 | | | | -1029.11 | | | 2074.42 | | | | | | 0.05 | | | | 0.30 | | | |  | | | |  | | |  |  |  |
| 2,3,4,6,7 | | 7 | | | | -1030.29 | | | 2074.72 | | | | | | 0.35 | | | | 0.25 | | | |  | | | |  | | |  |  |  |
| 2,3,4,5,6,7 | | 8 | | | | -1029.82 | | | 2075.83 | | | | | | 1.46 | | | | 0.15 | | | |  | | | |  | | |  |  |  |
| Term codes | | | | | |  | | | |  | | | | |  | | | |  | | | |  | | | |  | | |  |  |  |
| BIO12 | | BIO13 | | | | BIO14 | | | | BIO15 | | | | | BIO16 | | | | BIO17 | | | | BIO18 | | | | BIO19 | | |  |  |  |
| 1 | | 2 | | | | 3 | | | | 4 | | | | | 5 | | | | 6 | | | | 7 | | | | 8 | | |  |  |  |
| Model-averaged coefficients | | | | | | | | | |  | | | | |  | | | |  | | | |  | | | |  | | |  |  |  |
| (full average) | | | | | |  | | | |  | | | | |  | | | |  | | | |  | | | |  | | |  |  |  |
|  | | Estimate | | | | S.E | | | | Adj. Error | | | | | z value | | | | Pr(>\|z\|) | | | |  | | | |  | | |  |  |  |
| Intercept | | -1.46E-15 | | | | 3.36E-02 | | | | | | 3.37E-02 | | | 0 | | | | 1 | | | |  | | | |  | | |  |  |  |
| **BIO12** | | **1.79E-01** | | | | **2.01E-01** | | | | | | **2.06E-01** | | | **0.891** | | | | **0.372** | | | |  | | | |  | | |  |  |  |
| **BIO13** | | **4.38E-01** | | | | **1.79E-01** | | | | | | **1.79E-01** | | | **2.440** | | | | **0.014** | | | |  | | | |  | | |  |  |  |
| **BIO14** | | **1.37E+00** | | | | **3.16E-01** | | | | | | **3.17E-01** | | | **4.326** | | | | **1.52E-05** | | | |  | | | |  | | |  |  |  |
| **BIO15** | | **-9.59E-01** | | | | **2.09E-01** | | | | | | **2.09E-01** | | | **4.575** | | | | **4.80E-06** | | | |  | | | |  | | |  |  |  |
| **BIO17** | | **-2.26E+00** | | | | **3.43E-01** | | | | | | **3.43E-01** | | | **6.570** | | | | **<2E-16** | | | |  | | | |  | | |  |  |  |
| **BIO18** | | **-1.88E-01** | | | | **7.44E-02** | | | | | | **7.45E-02** | | | **2.533** | | | | **0.011** | | | |  | | | |  | | |  |  |  |
| **BIO19** | | **-1.10E-01** | | | | **7.65E-02** | | | | | | **7.66E-02** | | | **1.438** | | | | **0.150** | | | |  | | | |  | | |  |  |  |
| BIO16 | | 2.28E-01 | | | | 2.37E-01 | | | | | | 2.37E-01 | | | 0.960 | | | | 0.337 | | | |  | | | |  | | |  |  |  |
| (conditional average) | | | | | |  | | | |  | | | | | | | | |  | | | | | | | |  | | |  |  |  |
|  | | Estimate | | | | S.E | | | | Adj. Error | | | | | z value | | | | Pr(>\|z\|) | | | |  | | | |  | | |  |  |  |
| Intercept | | -1.46E-15 | | | | 3.36E-02 | | | | 3.37E-02 | | | | | 0 | | | | 1 | | | |  | | | |  | | |  |  |  |
| **BIO12** | | **2.99E-01** | | | | **1.78E-01** | | | | **1.78E-01** | | | | | **1.682** | | | | **0.092** | | | |  | | | |  | | |  |  |  |
| **BIO13** | | **4.38E-01** | | | | **1.76E-01** | | | | **1.80E-01** | | | | | **2.440** | | | | **0.014** | | | |  | | | |  | | |  |  |  |
| **BIO14** | | **1.37E+00** | | | | **3.16E-01** | | | | **3.17E-01** | | | | | **4.326** | | | | **1.52E-05** | | | |  | | | |  | | |  |  |  |
| **BIO15** | | **-9.59E-01** | | | | **2.09E-01** | | | | **2.09E-01** | | | | | **4.575** | | | | **4.80E-06** | | | |  | | | |  | | |  |  |  |
| **BIO17** | | **-2.25E+00** | | | | **3.43E-01** | | | | **3.43E-01** | | | | | **6.570** | | | | **<2E.16** | | | |  | | | |  | | |  |  |  |
| **BIO18** | | **-1.88E-01** | | | | **7.44E-02** | | | | **7.45E-02** | | | | | **2.533** | | | | **0.011** | | | |  | | | |  | | |  |  |  |
| **BIO19** | | **-1.10E-01** | | | | **7.65E-02** | | | | **7.66E-02** | | | | | **1.438** | | | | **0.150** | | | |  | | | |  | | |  |  |  |
| BIO16 | | 2.28E-01 | | | | 2.37E-01 | | | | 2.37E-01 | | | | | 0.960 | | | | 0.337 | | | |  | | | |  | | |  |  |  |
