# Supplementary material for: Direct development in Atlantic Forest anurans: What can environmental and biotic influences explain about its evolution and occurrence?
Source: PLoS One. 2023 Nov 30;18(11):e0291644. doi: 10.1371/journal.pone.0291644 (PMC10688756; doi:10.1371/journal.pone.0291644)
Supplement: S5 File — Direct and indirect effects of predictor variables on proportion of direct developing species of Atlantic Forest anuran communities. (DOCX) [file pone.0291644.s005.docx]

**S5 File – Result of piecewiseSEM model. Direct and indirect effects of predictor variables on proportion of direct developing species of Atlantic Forest anuran communities.** Goodness of fit: Fischer’s C = 2.635; P-value = 0.268; DF =2.Standard error (St. Error); degrees of freedom (DF); critical value (Crit. Value); and standardized estimate (St. Estimate)

| **Response** | **Predictor** | **Estimate** | **St. Error** | **DF** | **Crit.Value** | **P. Value** | **St. Estimate** |
| --- | --- | --- | --- | --- | --- | --- | --- |
| **PCPS1** | **BIO4** | **-9.26E-02** | **0.038** | **766** | **-2.421** | **0.016** | **-0.092** |
| PCPS1 | BIO12 | 5.86E-02 | 0.036 | 766 | 1.616 | 0.107 | 0.059 |
| **PCPS1** | **ET0** | **-4.19E-01** | **0.041** | **766** | **-10.261** | **0.000** | **-0.419** |
| **SES.FD** | **PCPS1** | **-3.23E-01** | **0.037** | **766** | **-8.740** | **0.000** | **-0.323** |
| SES.FD | BIO4 | 2.13E-02 | 0.060 | 766 | 0.358 | 0.721 | 0.021 |
| **SES.FD** | **BIO12** | **1.30E-01** | **0.054** | **766** | **2.393** | **0.017** | **0.130** |
| SES.FD | ET0 | -5.17E-02 | 0.077 | 766 | -0.671 | 0.502 | -0.052 |
| **SES.FD** | **Slope** | **2.09E-01** | **0.044** | **766** | **4.715** | **0.000** | **0.208** |
| **PROP.DD** | **SES.FD** | **2.31E-01** | **0.029** | **766** | **8.049** | **0.000** | **0.231** |
| **PROP.DD** | **PCPS1** | **-1.75E-01** | **0.031** | **766** | **-5.637** | **0.000** | **-0.175** |
| **PROP.DD** | **BIO4** | **-3.05E-01** | **0.063** | **766** | **-4.872** | **0.000** | **-0.305** |
| PROP.DD | BIO12 | 0.0847 | 0.061 | 766 | 1.383 | 0.167 | 0.085 |
| **PROP.DD** | **Slope** | **0.1612** | **0.040** | **766** | **4.041** | **0.000** | **0.161** |
| **PROP.DD** | **ET0** | **-0.4512** | **0.082** | **766** | **-5.492** | **0.000** | **-0.451** |

**Endogenous variable R² (R-squared).**

| **Response** | **R-squared** |
| --- | --- |
| PCPS1 | 0.17 |
| SES.FD | 0.13 |
| PROP.DD | 0.36 |
