# Supplementary material for: Direct development in Atlantic Forest anurans: What can environmental and biotic influences explain about its evolution and occurrence?
Source: PLoS One. 2023 Nov 30;18(11):e0291644. doi: 10.1371/journal.pone.0291644 (PMC10688756; doi:10.1371/journal.pone.0291644)
Supplement: S2 Appendix — (PDF) [file pone.0291644.s007.pdf]

S1 APPENDIX – Phylogenetic distribution of the development mode of 464 anurans from Atlantic Forest analyzed in the study. Blue rectangles indicate larval development (biphasic) and red rectangles indicate direct development. Species relationships are based on the consensus phylogeny of Jetz and Pyron (2018)

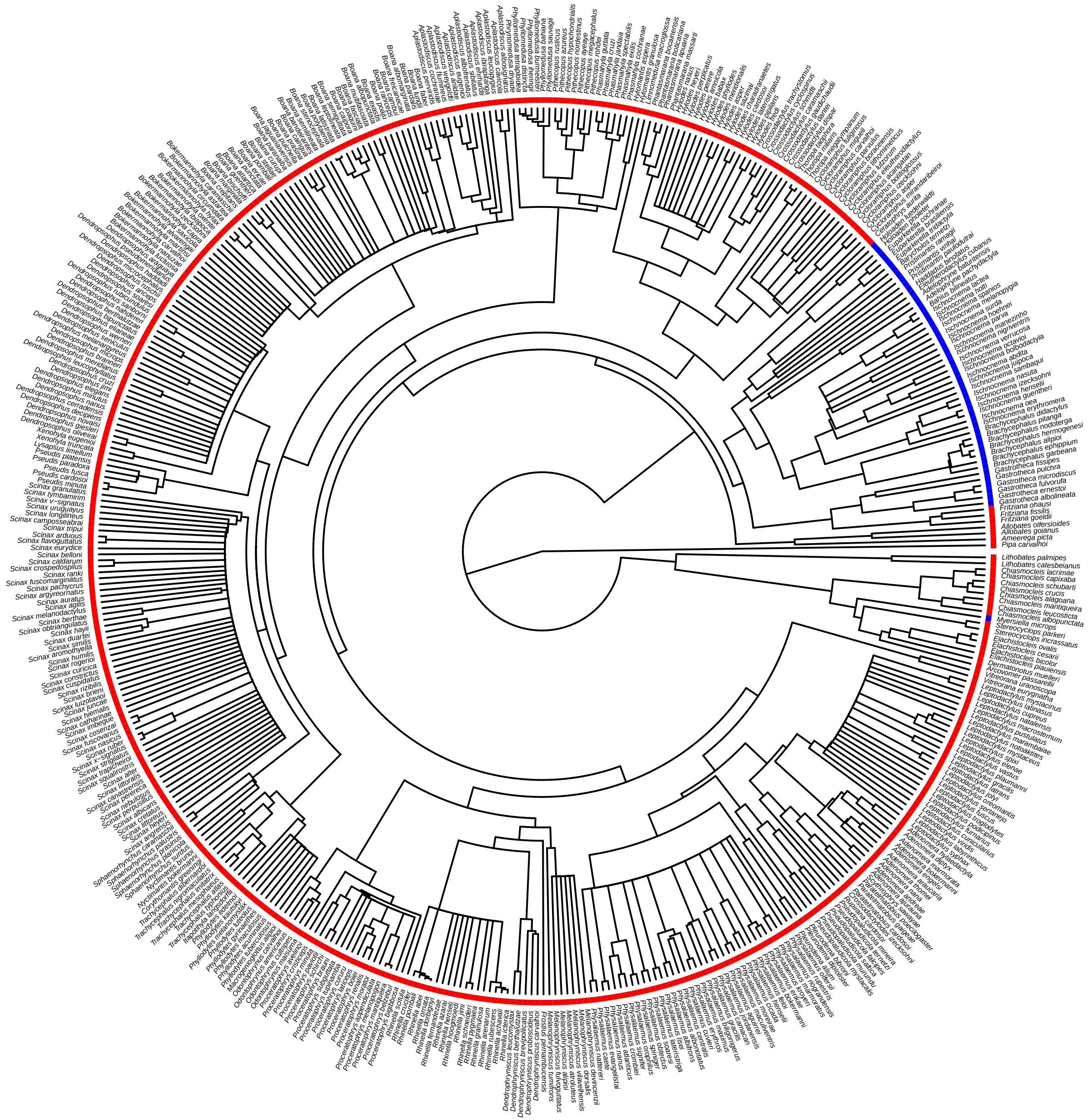
Pristimantis ramagii
Pristimantis vinhai
Pristimantis paulodutrai
Haddadus binotatus
Eleutherodactylus cubanus
Adelophryne baturitensis
Adelophryne pachydactyla
Bahius bilineatus
Ischnocnema lactea
Ischnocnema holti
Ischnocnema spanios
Ischnocnema melanopygia
Ischnocnema parva
Ischnocnema surda
Ischnocnema hoehnei
Ischnocnema manezinho
Ischnocnema nigriventris
Ischnocnema verrucosa
Ischnocnema octavioi
Ischnocnema bolbodactyla
Ischnocnema abdita
Ischnocnema juipoca
Ischnocnema sambaqui
Ischnocnema nasuta
Ischnocnema izecksohni
Ischnocnema henselii
Ischnocnema guentheri
Ischnocnema oea
Ischnocnema erythromera
Brachycephalus didactylus
Brachycephalus pitanga
Brachycephalus nodoterga
Brachycephalus hermogenesi
Brachycephalus alipioi
Brachycephalus ephippium
Brachycephalus garbeana
Gastrotheca fissipes
Gastrotheca pulchra
Gastrotheca microdiscus
Gastrotheca fulvorufa
Gastrotheca ernestoi
Gastrotheca albolineata
Fritziana ohausi
Fritziana fissilis
Fritziana goeldii
Allobates olfersioides
Allobates goianus
Ameerega picta
Pipa carvalhoi
Lithobates palmipes
Lithobates catesbeianus
Chiasmocleis lacrimae
Chiasmocleis capixaba
Chiasmocleis schubarti
Chiasmocleis crucis
Chiasmocleis alagoana
Chiasmocleis mantiqueira
Chiasmocleis leucosticta
Chiasmocleis albopunctata
Myersiella microps
Stereocyclops parkeri
Stereocyclops incrassatus
Elachistocleis ovalis
Elachistocleis cesarii
Elachistocleis bicolor
Elachistocleis piauiensis
Dermatonotus muelleri
Arcovomer passarellii
Vitreorana uranoscopa
Vitreorana eurygnatha
Leptodactylus mystacinus
