# Supplementary material for: Direct development in Atlantic Forest anurans: What can environmental and biotic influences explain about its evolution and occurrence?
Source: PLoS One. 2023 Nov 30;18(11):e0291644. doi: 10.1371/journal.pone.0291644 (PMC10688756; doi:10.1371/journal.pone.0291644)
Supplement: S3 Appendix — (DOCX) [file pone.0291644.s008.docx]

**S3 Appendix**


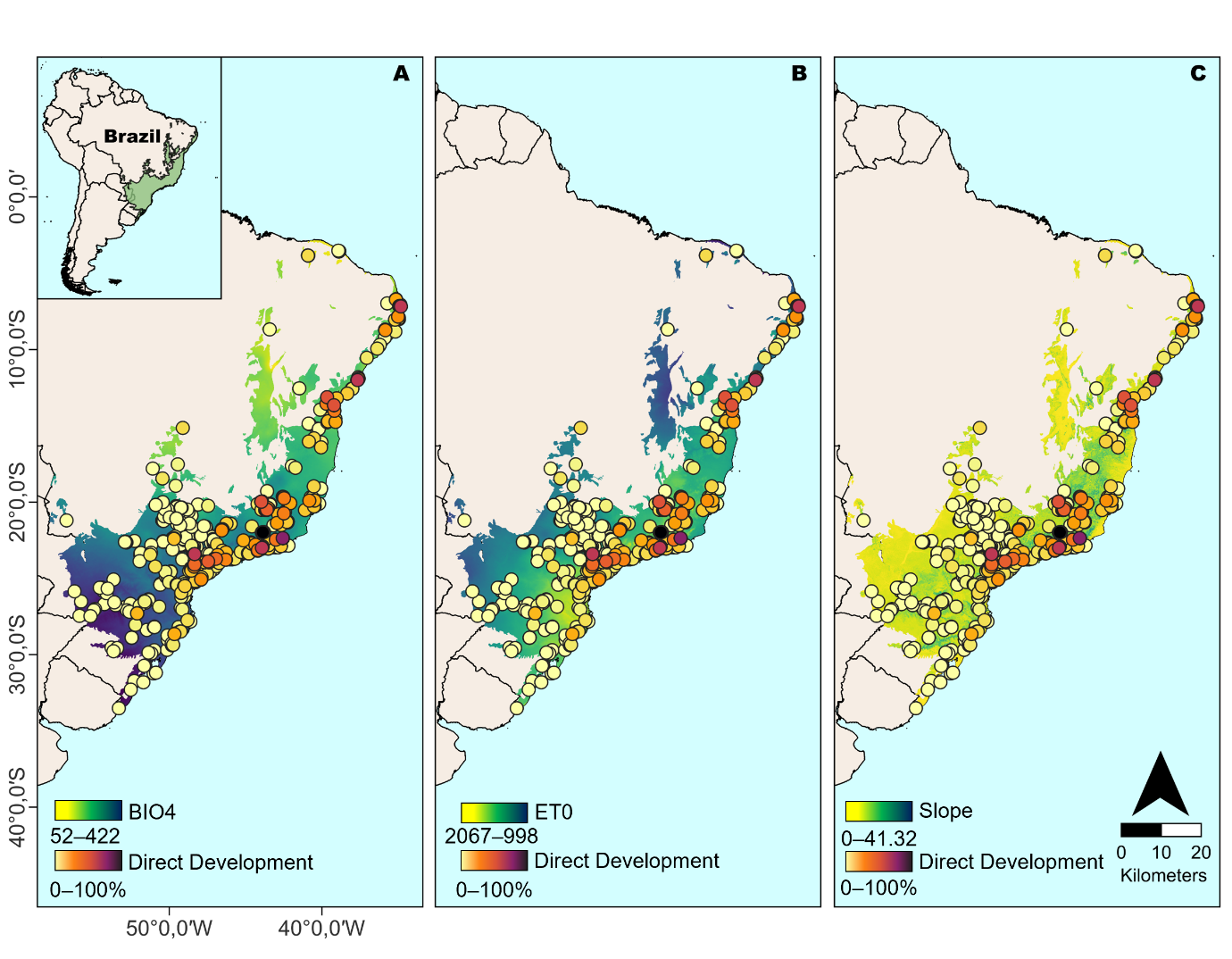


**Fig S1. Variation in the proportion of direct developing species in anuran Atlantic Forest communities and the environment.** A – temperature seasonality; B – potential evapotranspiration; and C – terrain slope. Base layer sources: South America shape file retrieved from the Database of Global Administrative Areas (GADM) under an open license (CC-BY): https://gadm.org/license.html; Atlantic Forest limits shape file from Muylaert et al. 2018 [34] under the GNU General Public License (https://github.com/LEEClab/ATLANTIC-limits/blob/master/LICENSE); communities (points) distributions were mapped based on records retrieved from Vancine et al. 2018 [30]; raster layers sources: temperature seasonality (BIO4) from WorldClim [35] (https://www.worldclim.org/data/worldclim21.html); ET0 from Global Aridity Index and Potential Evapotranspiration Climate Database [36] (https://figshare.com/articles/dataset/Global_Aridity_Index_and_Potential_Evapotranspiration_ET0_Climate_Database_v2/7504448/3) under license CC BY 4.4; and Slope from EarthEnv database [37]. Modified from Vancine et al. 2018 [30].


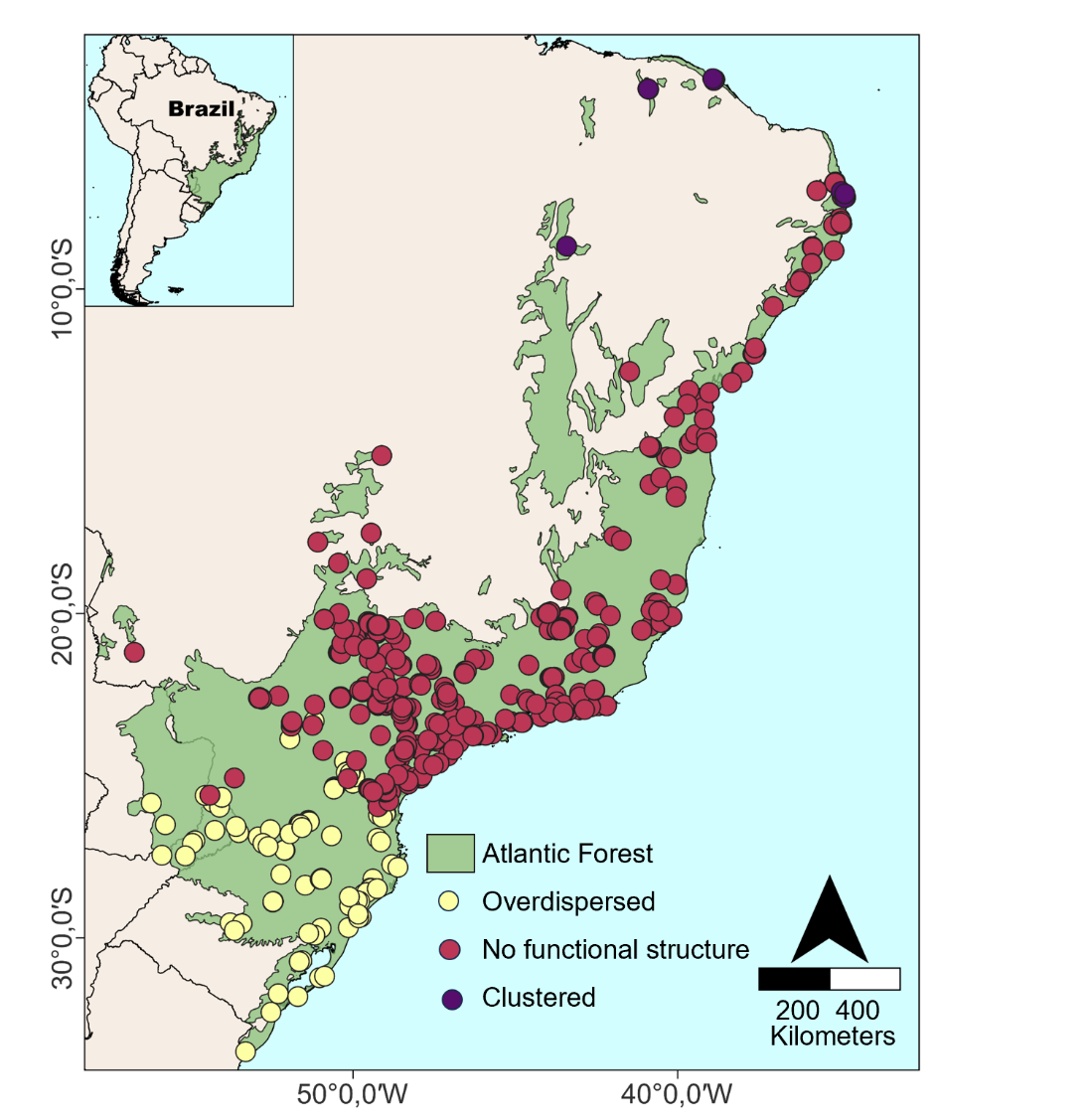


**Fig S2. Pattern of functional structure in anuran communities from the Atlantic Forest.** Light yellow points represent communities with FD values (functional diversity) lower than expected (functional overdispersion), dark purple points represent communities with FD values higher than expected (functional clustering), and magenta points represent communities with random functional structure. Base layer sources: South America shape file retrieved from the Database of Global Administrative Areas (GADM) under an open license (CC-BY): https://gadm.org/license.html; Atlantic Forest limits shape file from Muylaert et al. 2018 [34] under the GNU General Public License (https://github.com/LEEClab/ATLANTIC-limits/blob/master/LICENSE); communities (points) distributions were mapped based on records retrieved from Vancine et al. 2018 [30]. Modified from Vancine et al. 2018 [30].
